# Combined laparoscopic lymphoadenectomy of lateral pelvic and inguinal nodal metastases using indocyanine green fluorescence imaging guidance in low rectal cancer after preoperative chemoradiotherapy: a case report

**DOI:** 10.1186/s12876-022-02193-1

**Published:** 2022-03-16

**Authors:** Yanwu Sun, Yu Lin, Zhun Liu, Weizhong Jiang, Pan Chi

**Affiliations:** 1 Department of Colorectal Surgery and General Surgery, Fujian Medical University, Union Hospital, 29 Xinquan Road, Fuzhou, 350001 Fujian People’s Republic of China; 2 Department of Colorectal Surgery and General Surgery, Fujian Medical University, Union Hospital, Fuzhou, Fujian People’s Republic of China; 3 Minimal Invasive Center, Fujian Medical University Union Hospital, Fuzhou, Fujian People’s Republic of China; 4 Fujian Medical University, Fuzhou, Fujian People’s Republic of China

**Keywords:** Rectal cancer, Laparoscopic surgery, Inguinal lymph node metastasis, Lateral pelvic lymph node metastasis, Indocyanine green

## Abstract

**Background:**

Intraoperative near-infrared fluorescence (NIR) imaging with indocyanine green (ICG) can demonstrate real-time lymphatic drainage and thus improve the accuracy and completeness of lymphadenectomy in colorectal cancer surgery. However, it has not been utilized in the inguinal lymphadenectomy in rectal cancer. This study aimed to describe a case of combined laparoscopic lymphadenectomy of left lateral pelvic and inguinal nodal metastases using NIR imaging with ICG imaging guidance for a rectal cancer patient with left lateral pelvic and inguinal lymph node metastases.

**Case presentation:**

A 26-year-old man presented rectal cancer located 7 cm from the anal verge and enlarged lymph nodes in the left inguinal area. Pretreatment workup revealed rectal cancer with left lateral pelvic and inguinal lymph node metastases. The patient received preoperative chemoradiotherapy (pCRT), including radiation (total dose of 50.4 Gy in 28 fractions) to the whole pelvis and bilateral inguinal regions together with eight cycles of FOLFOX (oxaliplatin, fluoropyrimidine, and leucovorin) and three cycles of bevacizumab targeted chemotherapy. After pCRT, both colonoscopy and MR scan revealed a significant response of the primary tumor to pCRT, while MR scan revealed enlarged left lateral pelvic and inguinal lymph nodes. After four months from the completion of radiation (2 months after the last course of bevacizumab targeted therapy), the patient underwent laparoscopic-assisted ultra-low anterior resection and lymphadenectomy of left lateral pelvic and inguinal nodal metastases using ICG-NIR fluorescence imaging. The combined procedure was performed successfully without perioperative complication. Total operative time was 480 min and estimated blood loss 50 mL. Totally 34 lymph nodes were retrieved.

**Conclusions:**

This is the first report of the safety and feasibility of ICG-NIR fluorescence imaging-guided laparoscopic lymphadenectomy of left lateral pelvic and inguinal nodal metastases in managing low rectal cancer with lateral pelvic and inguinal LNs metastases.

**Supplementary Information:**

The online version contains supplementary material available at 10.1186/s12876-022-02193-1.

## Background

Inguinal lymph node metastasis (ILNM) occurs in approximately 1.2%-2.4% of rectal cancer patients with a dismal prognosis [1–3]. The TNM 8th edition staging system classifies rectal cancer with ILNM as distant metastasis that heralds a poor prognosis [4]. Inguinal lymph node dissection (ILND) is an integral part of the treatment of rectal cancer with ILNM [5] and can be beneficial for certain patients [6, 7]. Traditional ILND via an open approach is limited by high postoperative complications and impaired quality of life [8]. To minimize surgical morbidities, video endoscopic inguinal lymphadenectomy (VEIL) has been proposed as an alternative to open ILND in urological and gynecological surgeries [9].

Intraoperative near-infrared fluorescence (NIR) imaging with indocyanine green (ICG) can demonstrate real-time lymphatic drainage and thus improve the accuracy and completeness of lymphadenectomy in colorectal cancer surgery [10, 11]. It has also been reported to be a feasible technique to guide lateral pelvic lymph node dissection (LPLND) during rectal cancer surgery, which may help identify metastatic LNs and improve the surgical quality of lymphadenectomy [12, 13]. We hypothesized that ICG fluorescence imaging also has the potential to guide inguinal lymphadenectomy in rectal cancer. However, incorporating NIR/ICG fluorescence imaging guidance in VEIL for rectal cancer with ILNM has not yet been reported.

Herein, we presented a case of combined laparoscopic lymphadenectomy of left lateral pelvic and inguinal nodal metastases using NIR/ICG fluorescence imaging guidance for a rectal cancer patient with left lateral pelvic and inguinal lymph node metastases.

## Case presentation

**Fig. 1:**
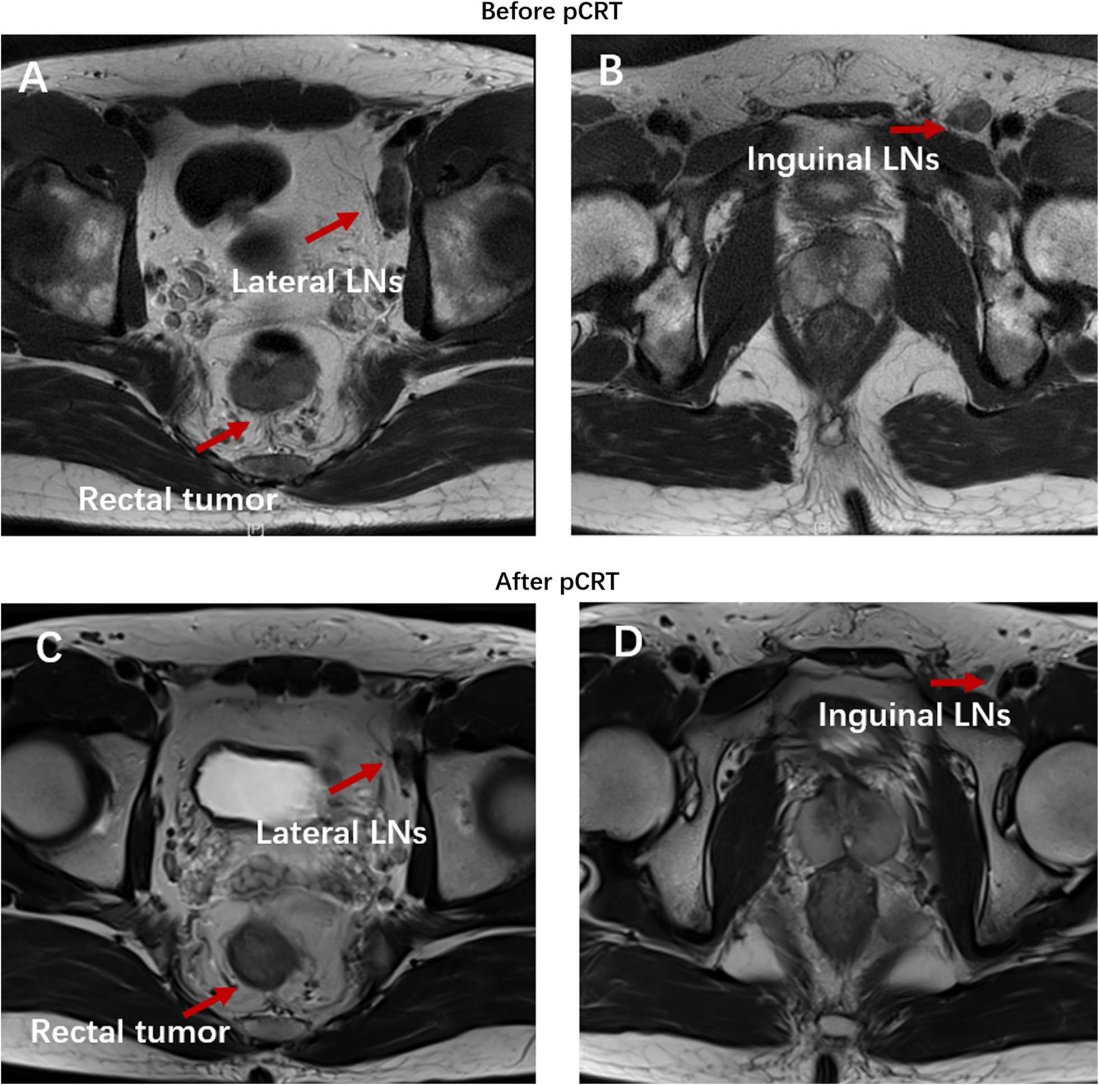
Pelvic MRI findings before and after pCRT in the high-resolution T2w sequences using a 3.0 Tesla Siemens Prisma MRI Scanner (Siemens Healthineers AG, Erlangen, Germany) with a contrast of Gadobutrol Injection (Gadovist). **A** Pelvic MRI before pCRT showing rectal tumor and left enlarged lateral LNs (axial T2WI sequence, no fat suppression, oblique transverse section, TR 2000, TE 95, FOV 20×20); **B** Pelvic MRI before pCRT showing left enlarged inguinal LNs (axial T2WI sequence, no fat suppression, oblique transverse section,TR 2000, TE 95, FOV 20×20); **C** After pCRT the rectal tumor and left enlarged lateral LNs decreased in size (axial T2WI sequence, no fat suppression, oblique transverse section, TR 2000, TE 89, FOV 22×22); **D** After pCRT the left enlarged inguinal LNs decreased in size (axial T2WI sequence, no fat suppression, oblique transverse section, TR 2000, TE 89, FOV 22×22). *MRI*: magnetic resonance imaging; *LNs*: lymph nodes; *pCRT*: preoperative chemoradiotherapy; *T2WI*: T2 weighted imaging; *TR*: repetition time; *ET*: echo time; *FOV*: field of view

A 26-year-old young man with low rectal cancer without family history was admitted in August 2020 with a complaint of rectal bleeding. Physical examination revealed a rectal mass located 7 cm from the anal verge (by digital rectal examination) and enlarged lymph nodes (LNs) in the left groin area. Colonoscopy demonstrated a cauliflower-shaped tumor located 7 cm from the anal verge, confirmed moderately differentiated adenocarcinoma on biopsy. Laboratory findings demonstrated elevated carcinoembryonic antigen (CEA) and carbohydrate antigen 19–9 (CA 19–9) levels (92.9 ng/mL and 146.8 U/mL, respectively). Magnetic resonance (MR) scan revealed a rectal tumor with irregularly shaped and enlarged lymph nodes in the left lateral pelvic (1.8 × 0.6 cm) and the left inguinal (2.0 × 1.5 cm) region, as depicted in Fig. 1A, B (MRI sequence can be seen in Additional file 3: Fig. S1). Furthermore, positron emission tomography/computed tomography (PET/CT) scan also demonstrated left lateral pelvic and inguinal lymph node metastasis. According to the multidisciplinary team's decision, the patient received preoperative chemoradiotherapy (pCRT), including radiation (total dose of 50.4 Gy in 28 fractions) to the whole pelvis and bilateral inguinal regions, as well as 8 cycles of FOLFOX (oxaliplatin, fluoropyrimidine, and leucovorin) and 3 cycles of bevacizumab targeted chemotherapy. After pCRT, MR scan revealed a significant response of the primary tumor to CRT, as shown in Fig. 1C, D. However, MR scan revealed enlarged left lateral pelvic (0.7 × 0.5 cm) and inguinal (1.4 × 0.8 cm) lymph nodes, as shown in Fig. 1C, D. PET/CT scan demonstrated hypermetabolism of 18F-fluorodeoxyglucose into both primary tumor (SUV max 1.0) and the left swollen lateral pelvic (SUV max 3.0) and inguinal lymph nodes (SUV max 3.4). In addition, the serum levels of CEA and CA199 have decreased dramatically (8.8 ng/mL and 21.8 U/mL, respectively).

After four months from the completion of radiation (2 months after the last course of bevacizumab targeted therapy), the patient underwent laparoscopic-assisted ultra-low anterior resection according to the principle of total mesorectal excision (TME). The patient was placed in the Trendelenburg semi-right lateral position under general anesthesia. After a lateral-to-medial approach of sigmoid colon mobilization, the inferior mesenteric artery was highly ligated. The splenic flexure was mobilized to ensure a tension-free anastomosis. Pelvic autonomic nerves were identified and preserved when mobilizing the rectum down to the pelvic floor.

Then, the rectum was transected 2 cm below the lower edge of the tumor. The specimen extraction was extracted via the stoma site. The proximal colon was transected 10 cm proximal to the tumor.

After completion of TME, NIR/ICG fluorescence imaging was used via a near-infrared camera system (KARL STORZ SE & Co. KG, Tuttlingen, Germany) to guide laparoscopic LLND (Additional file 1: Video 1). Two ml of ICG was injected around the tumor transanally 1 day before the operation. Using a fascia priority approach, the ureterohypogastric nerve fascia along the ureter was isolated, and the pelvic plexus was identified and preserved. The vesicohypogastric fascia along the internal iliac artery was isolated, and the parietal pelvic floor, such as the iliopsoas muscle and internal obturator muscle, was exposed. Next, the internal iliac LNs between the ureterohypogastric nerve fascia and the medial aspect of the vesicohypogastric fascia were dissected. An enlarged LN around the external iliac vein found positive in NIR/ICG fluorescence imaging was also separated from the external iliac vein and internal obturator muscle, and the obturator nerve was preserved. The obturator lymph nodes between the vesicohypogastric fascia and external iliac vessels were dissected. The residual soft tissues were examined by NIR/ICG fluorescence imaging to prevent the omission of LNs during LLND. After completion of LPLND, the uterus, the pelvic plexus, the internal and external iliac vessels and their branches, and the obturator nerve remained. Then, an end-to-end anastomosis was constructed using a circular stapler. A diverting ileostomy was performed to protect low rectal anastomosis.

**Fig. 2:**
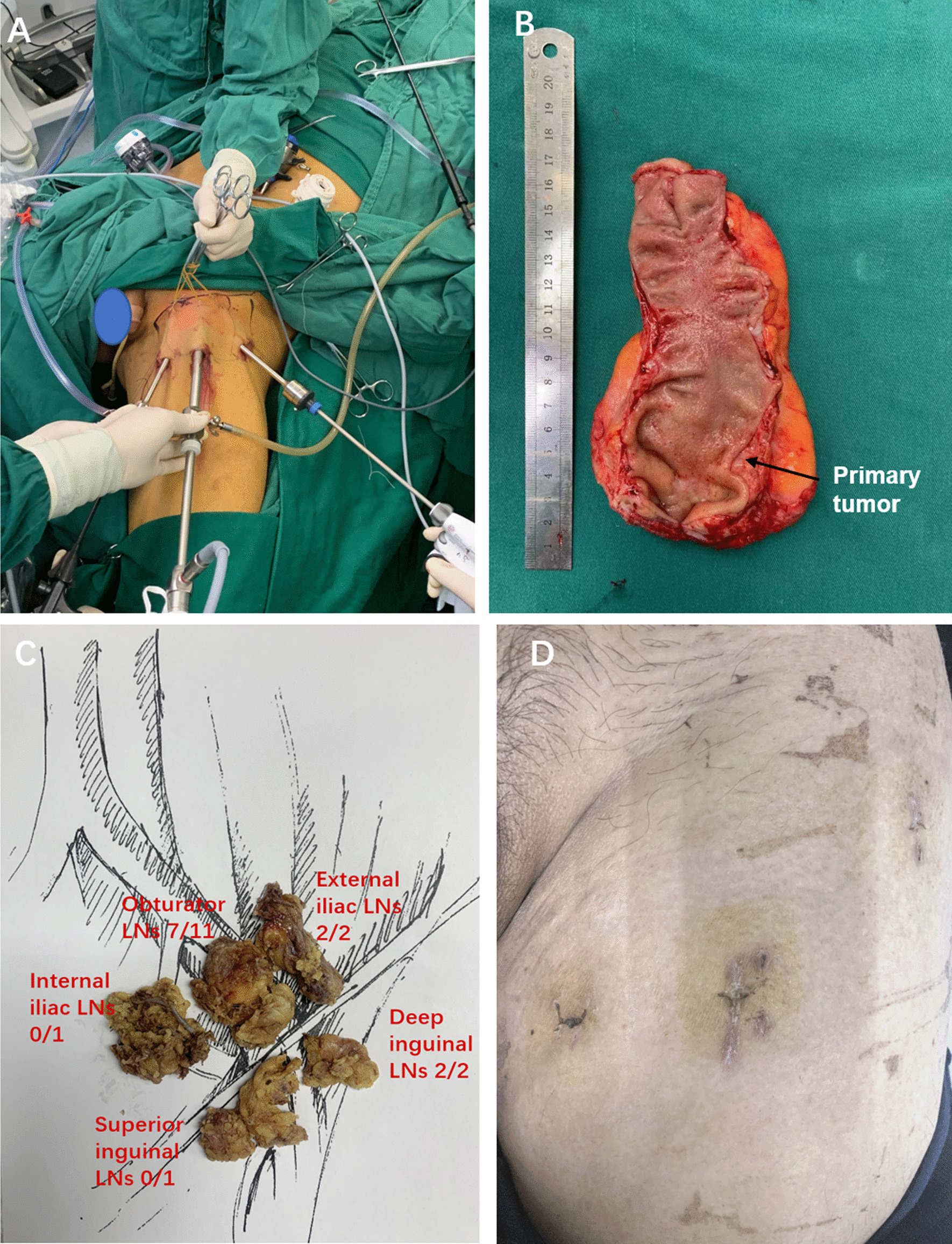
Intraoperative and pathological imaging of inguinal lymphadenectomy. **A** Trocar placement of left inguinal lymphadenectomy; **B** Gross appearance of the resected rectal tumor; **C** The resected left lateral and inguinal LNs. *pCRT*: preoperative chemoradiotherapy; *LNs*: lymph nodes

Next, the patient was switched to a supine position with thigh abduction. First, a 1.5 cm incision was made 2 cm below the lower vertex of the femoral triangle. NIR/ICG guided VEIL was performed using three ports, as shown in Fig. 2A. Scissors and digital maneuvers created a working space superficial to Scarpa’s fascia and then insufflated with CO2 at 12 mm Hg to maintain a visualization. The assistant grabbed the skin and lifted it to provide traction (Fig. 2A). As seen in Additional file 2: Video 2, the dissection boundaries of VEIL, including the sartorius muscle (laterally), adductor longus muscle (medially), and the inguinal ligament (superiorly), were well visualized. Then, superficial inguinal LNs were separated from Scarpa’s fascia. The saphenous vein medially and the spermatic cord, and the external inguinal ring superomedially were also identified and preserved. The saphenous vein is spared when superficial inguinal LNs dissection superior to the Scarpa’s fascia. Deep inguinal LNs dissection was then carried inferomedially starting from the transected Saphenofemoral junction around the femoral vein. Then, NIR/ICG fluorescence imaging confirmed that there were no residual LNs. Finally, the resected LNs were packed in a specimen retrieval bag and then extracted through the 10 mm trocar, and a suction drain was placed through the access of the 5 mm trocar. The total operating time was 480 min, and the estimated blood loss was 50 mL.

The surgical specimens showed a 1.2 × 1.5 cm scar after pCRT. The pathology revealed no residual carcinoma in the rectum with no tumor involvement in the perirectal, intermediate, and principal LNs, as shown in Fig. 2B, C. The internal iliac LN showed no tumor involvement, while pathology revealed infiltrated 7 of 11 dissected obturator LNs and 2 of 2 external iliac LNs. Noteworthily, pathology revealed infiltrated 2 of 3 dissected deep inguinal LNs, while the superficial inguinal LN had no tumor involvement. The tumor was classified as ypT0N0M1 according to the TNM classification of the Union for International Cancer Control (UICC). No postoperative incisional and urinary dysfunctions were observed, and the patient was discharged on postoperative day 8. Until now, the patient received FOLFOX chemotherapy for 4 cycles and bevacizumab targeted chemotherapy for 3 cycles. Postoperative surveillance (6 months) revealed no locoregional or systemic tumor relapse.

## Discussion and conclusions

Currently, there is no consensus nor guideline regarding the treatment strategy of ILNM from rectal cancer due to the rarity of this disease [14]. However, some studies have demonstrated that isolated ILNM from rectal cancer represents a distinct entity with a favorable prognosis after curative surgical resection [6, 15]. ILNM usually develops in low rectal cancer located below the dentate line [1]. Considering rectal cancer located 6 cm from the anal verge, in this case, ILNM can be explained by retrograde nodal spread owing to the anterograde lymphatic blockade by advanced primary tumors [10]. Pretreatment work-up excluded evidence of distant metastases.

The patient in the present study was classified as rectal cancer with solitary lateral pelvic and inguinal LNs metastases, that is, an oligometastatic state rather than systemic metastasis. In this respect, we proposed that the patient should be treated with curative intent.

According to current guidelines, prophylactical radiation of bilateral inguinal areas is not recommended for all rectal cancer patients, mainly due to significant radiation-induced side effects [16]. In addition, few studies have demonstrated the efficacy of pCRT in ILNM from rectal cancer. In our daily practice, pCRT was indicated from patients with locally advanced rectal cancer. For local control and possible cure, pCRT (radiation dose of 50.4 Gy in 28 fractions with concomitant FOLFOX and bevacizumab chemotherapy) was administered to the patient in this case study. Results from postoperative pathological examination revealed a pathologically complete response of the primary tumor to pCRT, while the effects on lateral pelvic and inguinal LNs were not that pronounced. The discrepancy in treatment response of primary tumor and metastatic lateral pelvic and inguinal LNs suggested the treatment insufficiency of pCRT. It indicated the rationale for performing lymphadenectomy for both lateral pelvic and inguinal LNs metastasis.

Previous studies have demonstrated a survival benefit for rectal cancer surgery with isolated ILNM from rectal cancer [6, 15]. Aiming at decreasing postoperative morbidities of ILND, VEIL has been proposed as an alternative to traditional open ILND in urological and gynecological surgeries. Only one study has reported the utility of VEIL in the management of rectal cancer with ILNM [17]. Herein, the patient experienced an uneventful postoperative course without any complication, such as skin flap necrosis, wound dehiscence, femoral vessel and femoral nerve injury, deep vein thrombosis, and lymphocele [9]. Previous studies have demonstrated similar oncologic outcomes between VEIL and traditional open ILND in genitourinary cancer surgeries. In contrast, the oncological effect of VEIL in rectal cancer with ILNM remains unclear.

Recurrence of pelvic lateral LNs may be related to omission of LNs metastases during LPLND. NIR/ICG fluorescence imaging has been used as a tool to guide LPLND in rectal cancer surgery, which may assist in identifying metastatic LNs and potentially improve the quality of lymphadenectomy [12, 13]. Herein, NIR/ICG fluorescence imaging was used to guide LPLND and utilized to assist lymphadenectomy during VEIL. Our findings demonstrated that NIR/ICG fluorescence imaging-guided laparoscopic LPLND and VEIL was safe and feasible. Besides, ICG has the potential to guided VEIL in rectal cancer.

In conclusion, this study for the first time demonstrated the safety and feasibility of NIR/ICG fluorescence imaging-guided laparoscopic LPLND and VEIL in the management of low rectal cancer with lateral pelvic and inguinal LNs metastasis. NIR/ICG fluorescence imaging-guided laparoscopic LPLND and VEIL is a technically promising technique. Nevertheless, further prospective studies with longer follow-up are warranted to clarify the oncological efficacy of NIR/ICG fluorescence imaging-guided laparoscopic LPLND and VEIL in the treatment of rectal cancer.

## Supplementary Information

**Additional file 1: Video 1.** V1 LPLND 1.0. Video file of NIR/ICG fluorescence imaging guided laparoscopic LPLND.

**Additional file 2: Video 2.** V2 VEIL 0.9. Video file of NIR/ICG fluorescence imaging guided laparoscopic VEIL.

**Additional file 3: Fig. S1.** Original MRI images of Fig. 1 that contains sequences.

## Data Availability

The datasets generated and/or analyzed during the current study are not publicly available due to protecting individual patient privacy but are available from the corresponding author on reasonable request.

## Abbreviations

ILNM: Inguinal lymph node metastasis
ILND: Inguinal lymph node dissection
VEIL: Video endoscopic inguinal lymphadenectomy
NIR: Near-infrared fluorescence
ICG: Indocyanine green
LPLND: Lateral pelvic lymph node dissection
LNs: Lymph nodes
CEA: Carcinoembryonic antigen
CA 19-9: Carbohydrate antigen 19-9
PET/CT: Positron emission tomography/computed tomography
SUV: Standardized uptake value
pCRT: Preoperative chemoradiotherapy
FOLFOX: Oxaliplatin, fluoropyrimidine, and leucovorin
TME: Total mesorectal excision
