# Supplementary figures and images for: Combined laparoscopic lymphoadenectomy of lateral pelvic and inguinal nodal metastases using indocyanine green fluorescence imaging guidance in low rectal cancer after preoperative chemoradiotherapy: a case report
Source: BMC Gastroenterol. 2022 Mar 16;22:123. doi: 10.1186/s12876-022-02193-1 (PMC8925188; doi:10.1186/s12876-022-02193-1)

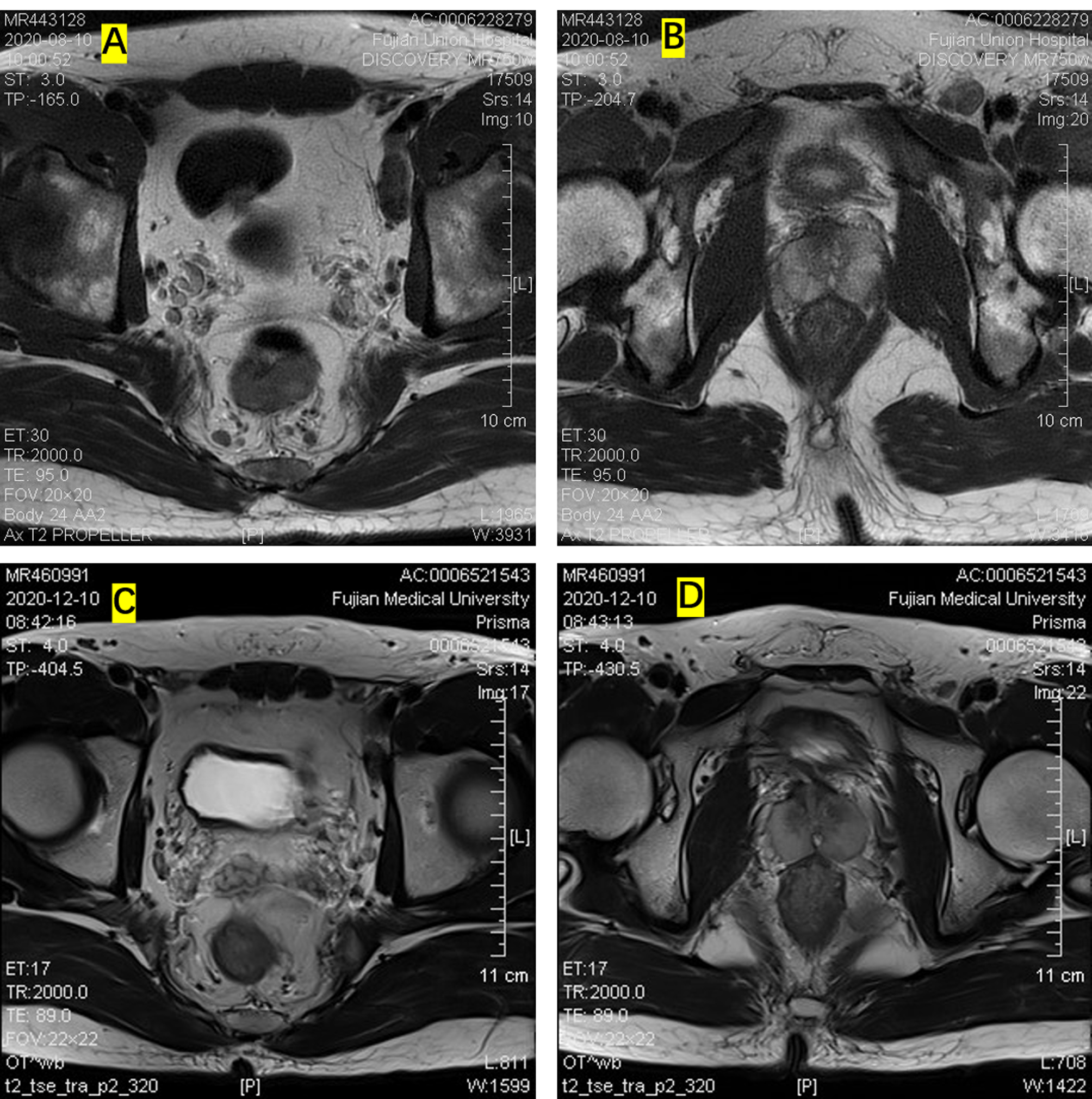

Supplement: Supplementary file 3 — Additional file 3: Fig. S1. Original MRI images of Fig. 1 that contains sequences. [file 12876_2022_2193_MOESM3_ESM.tif]
